# M. Payan on the Employment of the Ergot of Rye in Cases of Paraplegia

**Published:** 1842-10-08

**Authors:** 

**Affiliations:** Aix


*Employment of the Ergot of Rye in Cases of Paraphlegia.* By M. Payan, of Aix.—M. Payan begins by stating that he has recently sought to demonstrate that it was wrong to consider ergot of rye as a simple excitor of uterine contractility; that facts prove that the ergot acts on the rectum, the bladder, and the inferior extremities, when these parts are in certain asthenic conditions, in the same manner as on the womb in cases of uterine inertia; and that, not being able to attribute rationally to this agent specific effects on parts of the body essentially different, he was obliged to seek for its action still further, and to refer it to some organ holding still under its influence these different parts. M. Payan has discovered that it is on the spinal marrow that the ergot exercises a special and primitive action.

This fact being established, it became evident that we ought to recur to the use of the ergot in paraphlegias and debility of the inferior extremities, proceeding from causes which have suspended or weakened the action of the spinal marrow, without having altered its texture.

M. Payan adds the three following facts to those which he has already published, and which demonstrate the justice of his views:

1. A man of 40 years, fell on the perineum, and paraphlegia was the consequence. The patient, treated at Marseilles, was completely cured. Some time after, in a journey which he made to Aix, he again experienced the same symptoms, and entered the Hotel Dieu of that city. In the absence of M. Payan opiate liniments, blisters, &c., were unsuccessfully used against the paraphlegia. The case then came under M. Payan's care, who substituted for these means one gramme* of the ergot to be taken once. Twelve hours after the paralyzed limb became agitated frequently by muscular starts, and he daily recovered strength. At the end of six days the patient could walk with the aid of a single crutch. During a fortnight the ergot was administered in the dose of two grammes. A month after, the treatment having been suspended on the fifteenth day, on account of the appearance of some slight gastric derangement, the amelioration persisted, but the patient left the hospital.

2. A man of 30, attacked with a complete paraphlegia, committed himself to the care of M. Payan. A number of energetic measures had already been tried. This was his state when he was submitted to the observation of this skilful physician.

The two lower limbs support well enough the weight of the body; but he finds it impossible to walk any time without sitting down; unless he does so he infallibly falls. The right lower extremity is strong enough, but insensible; the other limb is sensible, but a little atrophied. The bladder has lost a part of its contractility.

A gramme of the ergot was administered every morning. At the end of a few days the dose was increased to two grammes. At the same time frictions with a stimulating liniment were made along the spine. After eighteen days the two lower extremities had become strong, and the patient could return into his country.

3. A workman, having endured a very severe paraphlegia, the consequence of a bad attack from lead; different means had failed to relieve the malady; the ergot completely triumphed over it.

From these three cases M. Payan not only infers the efficacy, but, more-

*Equivalent to 15 grs. English.

over, the complete innocence of this medicine, which has been ordered for six weeks without any accident, which this year has been given for forty consecutive days, in doses of forty to eighty centi-grammes, to a young girl five years old, without her being the least injured by it.—*Dub. Journ. of Med. Sci., from Journ. de Pharm.*

